# GCNG: graph convolutional networks for inferring gene interaction from spatial transcriptomics data

**DOI:** 10.1186/s13059-020-02214-w

**Published:** 2020-12-10

**Authors:** Ye Yuan, Ziv Bar-Joseph

**Affiliations:** 1 Machine Learning Department, School of Computer Science, Carnegie Mellon University, Pittsburgh, PA 15213 USA; 2 Computational Biology Department, School of Computer Science, Carnegie Mellon University, Pittsburgh, PA 15213 USA

**Keywords:** Spatial transcriptomics, Graph convolutional networks, Extracellular gene interactions

## Abstract

Most methods for inferring gene-gene interactions from expression data focus on intracellular interactions. The availability of high-throughput spatial expression data opens the door to methods that can infer such interactions both within and between cells. To achieve this, we developed Graph Convolutional Neural networks for Genes (GCNG). GCNG encodes the spatial information as a graph and combines it with expression data using supervised training. GCNG improves upon prior methods used to analyze spatial transcriptomics data and can propose novel pairs of extracellular interacting genes. The output of GCNG can also be used for downstream analysis including functional gene assignment.

Supporting website with software and data: https://github.com/xiaoyeye/GCNG.

## Background

Several computational methods have been developed over the last two decades to infer interaction between genes based on their expression [1]. Early work utilized large compendiums of microarray data [2] while more recent work focused on RNA-Seq and scRNA-Seq [3]. While the identification of pairwise interactions was the goal of several studies that relied on such methods, others used the results as features in a classification framework [4] or as pre-processing steps for the reconstruction of biological interaction networks [5]. Most work to date focused on intra-cellular interactions and network. In such studies, we are looking for interacting genes involved in a pathway or in the regulation of other genes within a specific cell. In contrast, studies of extracellular interactions (i.e., interactions of genes or proteins in different cells) mainly utilized small-scale experiments in which a number of ligand and receptor pairs were studied in the context of a cell line or tissue [6]. However, recently developed methods for spatial transcriptomics are now providing high-throughput information about both, the expression of genes within a single cell and the spatial relationships between cells [7–11]. Such information opens the door to much larger-scale analysis of extracellular interactions.

Current methods for inferring extracellular interactions from spatial transcriptomics have mostly focused on unsupervised correlation-based analysis. For example, the Giotto method calculated the effect upon gene expression from neighbor cell types [12]. While these approaches perform well in some cases, they may not identify interactions that are limited to a specific area, specific cell types, or that are related to more complex patterns (for example, three-way interactions).

To overcome these issues, we present a new method that is based on graph convolutional neural networks (GCNs). GCNs have been introduced in the machine learning literature a few years ago [13]. Their main advantage is that they can utilize the power of convolutional NN even for cases where spatial relationships are not complete [14, 15]. Specifically, rather than encoding the data using a 2D matrix (or a 1D vector), GCNs use the graph structure to encode relationships between samples. The graph structure (represented as a normalized interaction matrix) is deconvolved together with the information for each of the nodes in the graph leading to NN that can utilize both, the values encoded in each node (in our case gene expression) and the relationship between the cells expressing these genes.

To apply GCN to the task of predicting extracellular interactions from gene expression (GCNG), we first convert the spatial transcriptomics data to a graph representing the relationship between cells. Next, for each pair of genes, we encode their expression and use GCNG to convolve the graph data with the expression data. By this way, the NN can utilize not just first-order relationships, but also higher-order relationships in the graph structure. We discuss the specific transformation required to encode the graph and gene expression, how to learn parameters for the GCNG, and how to use it to predict new interactions.

We test our approach on three datasets from the two spatial transcriptomics methods that profile the most number of genes right now, SeqFISH+ [16] and MERFISH [17]. As we show, GCNG greatly improves upon correlation-based methods when trying to infer both autocrine and extracellular gene interactions involved in cell-cell interactions. We visually analyze some of the correctly predicted pairs and show that GCNG can overcome some of the limitations of unsupervised methods by focusing on only a relevant subset of the data. Analysis of the predicted genes shows that many are known to be involved in a similar functional pathway supporting their top ranking.

## Results

### The GCNG framework

**Fig. 1:**
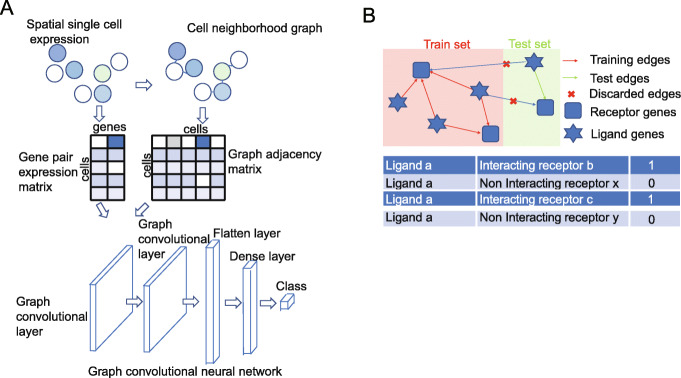
GCNG for extracellular gene relationship inference. **a** GCNG model using spatial single cell expression data. A binary cell adjacent matrix and an expression matrix are extracted from spatial data. After normalization, both matrices are fed into the graph convolutional network. **b** Training and test data separation and generation strategy. The known ligand and receptor genes can form complicated directed networks. For cross-validation, all ligand and receptors are separated exclusively as training and test gene sets, and only gene pairs where both genes are in training (test) gene set are used for training (test). To balance the dataset, each positive ligand-receptor (*L*_*a*_, *R*_*b*_) gene pair with label 1 will have a negative pair sample (*L*_*a*_, *R*_*x*_) with label 0 where *R*_*x*_ was randomly selected from all training (test) receptor genes which are not interacting with *L*_*a*_ in training (test)

We extended ideas from GCN [18, 19] and developed the Graph Convolutional Neural networks for Genes (GCNG), a general supervised computational framework for inferring gene interactions involved in cell-cell communication from spatial single cell expression data. Our method takes as input both, the location of the cells in the images and the expression of gene pairs in each of these cells. GCNG starts by representing single cell spatial expression data using two matrices. The first encodes cell locations as a neighborhood graph, while the second encodes the expression of genes in each cell. These two matrices are used as inputs for a five-layer graph convolutional neural network which aims to predict cell-cell communication gene relationships (Fig. 1a). The core structure of GCN is its graph convolutional layer, which enables it to combine graph structure (cell location and neighborhood) and node information (gene expression in specific cell) as inputs to a neural network. Since the graph structure links spatially proximal cells, GCNs can utilize convolutional layers that underly much of the recent success of neural networks, without directly using image data [14, 15]. Specifically, GCNG consists of two graph convolutional layers, one flatten layer, one 512-dimension dense layer, and one sigmoid function output layer for classification. Note that we are using two convolutional layers here allowing the method to learn indirect (i.e., non-physical or two-layer) graph relationships as well. Since the impact of regulatory proteins can be larger than just direct neighbors such an approach allows the method to infer interactions that may be missed by only considering direct neighbors. Training GCNG requires the use of positive and negative pairs and we discuss below the data we used to obtain such training samples. After training, GCNG can predict, for any pair of genes, whether they are interacting in the dataset being studied.

### Applying GCNG to spatial transcriptomics data

While a number of recent methods have been suggested for spatial profiling of single cell RNA-Seq data [7–11], we decided to focus on the two methods that currently provide expression levels for the most number of genes in such experiments. The first is seqFISH+ [16]. We tested two datasets that used seqFISH+. The first contained information on the expression of 10,000 genes in 913 cells in the mouse cortex and the second profiled 2050 cells in mouse olfactory bulb (OB) in seven separate fields of view. The second method we used is MERFISH [17] for which we analyzed a dataset consisting of 10,050 genes in 1368 cells. Unlike the seqFISH+ data that profiled the expression in-vivo, the MERFISH data is from in vitro cultured cells and so does not include a diverse set of cell types. Still, as the authors of the MERFISH paper observed, even within this population, there are differences in spatial expression and so the data can be used to study extracellular gene-gene interaction. We normalized the expression data such that expression levels for all genes in each cell sum to the same value as was previously done [16]. See the “Methods” section and Additional file 1 for complete details on both datasets.

GCN requires labeled data for supervised training. While the exact set of signaling interactions between cells in the spatial data we studied is unknown, we used as true interactions a curated list of interacting ligands and receptors [20]. Ligands are proteins that are secreted by cells and they then interact with membrane receptor proteins on the cell itself or on neighboring to activate signaling pathways within the receiving cell [21]. See the “Methods” section for complete details on the positive and negative pairs used for training.

For evaluation, GCNG adopted a tenfold cross-validation. Train and test sets were completely separated to avoid any information leakage (Fig. 1b). See the “Methods” section and Additional file 1 for details.

### GCNG correctly infers ligand-receptor interactions between cells

We first evaluated GCNG’s ability to predict ligand-receptor interactions. For this, we used two datasets. The first is seqFISH+ mouse cortex tissue which contains the expression of 10,000 genes in 913 cells. Our labeled set consisted of 1056 known interactions between 309 ligands and 481 receptors. The second is a MERFISH dataset with 10,050 genes from 1368 cells, and 841 known interactions between 270 ligands and 376 receptors.

We enforced a strict separation between the training and test sets in the 10-fold cross-validation (CV) (Fig. 1b). Negative pairs were also ligand-receptor and were randomly selected from non-interacting training (test) data genes. We also used the 10-fold CV to select hyper-parameters to determine the neighborhood for each cell (Methods). We compared three possible GCNG models: diagonal GCNG that only uses a diagonal matrix to represent the graph so that only autocrine interactions are possible, exocrine GCNG where only exocrine interaction between cells are allowed, and autocrine plus (+) GCNG that allows for both autocrine and exocrine interactions. To evaluate the performance of GCNG, we compared it to a number of prior methods that were recently used to predict genes involved in extracellular gene interactions from spatial expression data. These include computing the spatial Pearson correlation (PC) between ligand and receptors in neighboring cells and Giotto [12] which first calculates a similarity score for all pairs of genes in all pairs of neighboring cell types and we then rank pairs based on their average score.

We also compared GCNG to two alternative methods that do not use spatial information at all to determine the contribution of neighborhood data. These included Pearson’s correlation between the expression of ligand and receptors within each cell [22] and our diagonal GCNG method with only autocrine interactions. Finally, we compared GCNG performance on the real data to results when applied to permutation of both, the neighborhood information for each cell and the set of interacting ligand-receptors used for training and testing. We also tested additional variants of GCNG including variants that utilized cell type information (encoded as a node attribute), edge weight (using the distance between cells), and variants using other GNN architectures including EdgeconditionConv [23] and graph attention [24] (Additional file 1: Fig. S1).

**Fig. 2:**
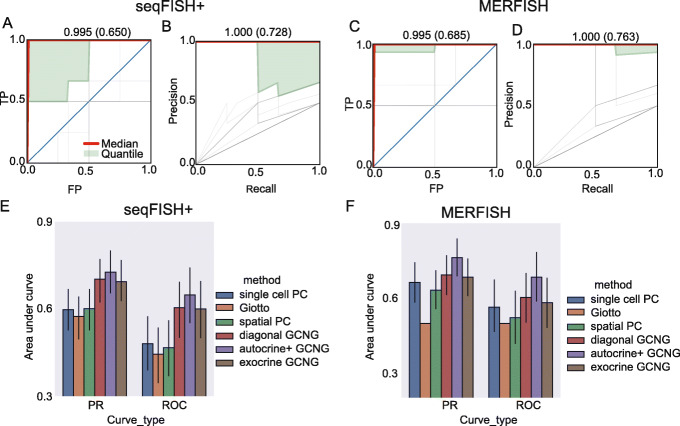
GCNG correctly infers extracellular ligand-receptor interaction. **a**, **b** AUROC and AUPRC curves for seqFISH+ using autocrine+ GCNG model. Here, each gray line represents results for one ligand (a total of 91 curves), red line represents the median curve, and the light green part represents the region between 40 and 60 quantile. Median and mean of area under the curves are shown on top of each panel. **c**, **d** AUROC and AUPRC curves for MERFISH when using the autocrine+ GCNG model (73 curves). **e**, **f** Overall comparison in terms of AUROC and AUPRC for single cell Pearson correlation, Giotto, spatial Pearson correlation, diagonal GCNG, autocrine+ GCNG, and exocrine GCNG models. For Giotto on MERFISH (**f**), we set its all AUROC and AUPRC as 0.5 since the data only has one cell type

Results are presented in Fig. 2. As can be seen, GCNG achieved the best results in both datasets (Fig. 2a–d). Specifically, for seqFISH+ cortex data, autocrine+, diagonal, and exocrine GCNG reached mean (median) AUROC/AUPRC of 0.65/0.73 (0.99/1.0), 0.59/0.70 (0.99/1.0), and 0.60/0.69 (0.99/1.0), respectively. In contrast, for this data spatial PC, Giotto and single cell PC all performed much worse with mean (median) AUROC/AUPRC of 0.54/0.65 (0.75/0.79), 0.45/0.58 (0.25/0.33), and 0.48/0.60 (0.38/0.38), respectively. For MERFISH data, autocrine+, diagonal, and exocrine GCNG reached mean (median) AUROC/AUPRC of 0.69/0.76 (0.99/1.0), 0.60/0.69 (0.99/1.0), and 0.61/0.71 (0.75/0.79), respectively, again improving on the other methods we compared to. See also Additional file 1: Figs. S2&3 for detailed performance values. Overall, for both datasets, GCNG achieves a relative improvement of at least 20% for mean AUROC/AUPRC when compared to prior methods. In addition, the fact that autocrine+ GCNG outperformed diagonal GCNG for both datasets confirms the importance of spatial information for this task.

To test if the interactions identified are likely active in the tissue tested, we further compared the performance when using the real interaction data to running GCNG on a permuted training and test dataset (in which we permute the set of interacting ligand-receptor pairs). We observed a large drop in performance when using the randomized data (autocrine plus GCNG in terms of mean AUROC for MERFISH (seqFISH+), real vs. random: 0.69 vs. 0.55 (0.65 vs. 0.52); exocrine GCNG vs. random: 0.58 vs. 0.50 (0.60 vs. 0.43). See Additional file 1: Fig. S1 for detailed comparison results on both datasets.

### Analysis of co-expression patterns identified by GCNG

**Fig. 3:**
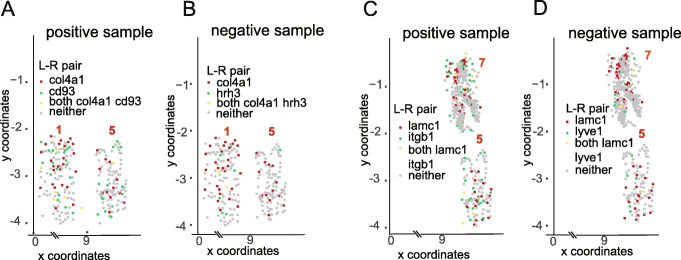
Spatial expression patterns for selected ligand-receptor pairs. **a**, **b** Spatial expression distribution of correctly predicted positive (cd93) and negative (hrh3) receptors for ligand col4a1. Two of the 7 fields of view (FOV) profiled are shown (see Additional file 1: Fig. S4 for all FOVs). As can be seen, the correctly predicted pairs are indeed much better spatially correlated than the negative pair. **c**, **d** Spatial expression distribution of correctly predicted positive (itgb1) and negative (lyve1) receptors for ligand lamc1. Cells highly expressing lamc1 and itgb1 are both concentrated in the 5th and 7th fields as shown

To further explore the predictions of our GCNG and to map them back to the original spatial representation, we looked at some of the top correctly predicted pairs. For each such pair (a ligand and receptor predicted to interact), we projected their expression on the cell distribution figures. Figure 3 presents such projection for two local ligands (col4a1 and lamc1) with their positive and negative receptor partners in seqFISH+ cortex data (Fig. 3a, b for col4a1, and c, d for lamc1) Since here the genes are fixed while cells need to be selected (in contrast to common cases where for each cell highly expressed genes are selected), for a gene, cells are defined to “highly expressing” it if the expression of the gene in that cell is in the top 100 expression levels for that gene among all cells. For the positive col4a1-cd93 pair, cells highly expressing col4a1 and cd93 are both concentrated in the 1st and 5th fields, which have the most cells highly expressing the ligand or receptor genes (see Additional file 1: Fig. S4A and B for plots of all fields of view). In contrast, for the negative col4a1-hrh3 pair, cells highly expressing hrh3 do not seem to reside next to cells expressing col4a1. Similar pattern comparison can also be observed for ligand lamc1 with positive (itgb1) and negative receptor (lyve1) (Fig. 3c, d (see Additional file 1: Fig. S4C and D for all fields of view)). The ability of GCNG to predict such interactions based on a subset of the data highlights the usefulness of this approach compared to global analysis methods including PC. Cell type plots (Additional file 1: Fig. S5) indicate that correctly predicted pairs can be found in both, neighboring cells from the same type and cells from different types. These results indicate that the GCNG method can generalize well and can be used to correctly identify several different types of interactions.

### Inferring causal interactions

**Fig. 4:**
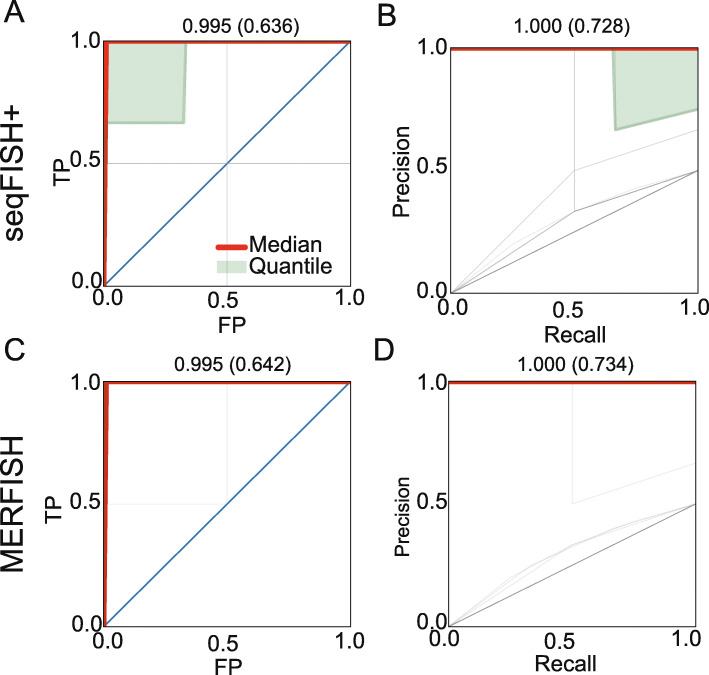
GCNG can infer the direction of extracellular ligand-receptor interactions. **a**, **b** AUROC and AUPRC of GCNG for the direction prediction task using the seqFISH+ dataset. **c**, **d** AUROC and AUPRC of GCNG for the direction prediction task using the MRFISH dataset

While correlation-based methods can be used to identify gene co-expression interactions and networks [25, 26], these methods cannot be used to infer causality since their outcome is symmetric. Causality information may be trivial for ligand receptors, since the direction for such pair is known. However, for other interacting genes across cells, the direction is often not clear. Thus, a method that can infer both interactions and directionality may be beneficial for studying spatial transcriptomics data. Unlike prior unsupervised methods, our supervised framework can be trained to identify causal interactions based on the pair-wise spatial expression pattern if training data exists, inspired by a recent work [27]. We thus trained a GCNG on a subset of known causal pairs (ligand and receptors) and then used it to predict directionality for other pairs. To generate train and test data for this, for each known ligand-receptor (*L*_*a*_, *R*_*b*_) gene pair, we introduce a negative sample (*R*_*b*_, *L*_*a*_) with label 0. The same tenfold cross-validation strategy is used to evaluate GCNG’s performance here. Results are presented in Fig. 4. As can be seen for seqFISH+ cortex and MERFISH datasets, GCNG performs well on this task with mean (median) AUROC/AUPRC of 0.636/0.728 (0.99/1.0) and 0.642/0.734 (0.99/1.0), respectively. Thus, for top predicted pairs, the direction predictions of GCNG can be used to further assign causality.

### Functional gene assignment

**Fig. 5:**
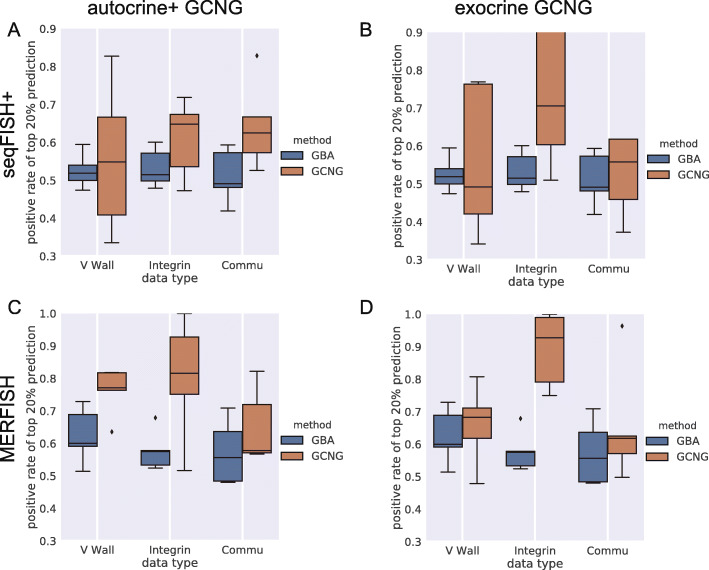
Function assignment. **a** Fraction of correctly assigned genes in top 20% of predictions for autocrine+ GCNG and GBA method for “integrin cell surface interactions,” “cell surface interactions at the vascular wall,” and “cell-cell communication” functions on seqFISH+ data. **b** Results for exocrine GCNG on seqFISH+ data. **c**, **d** Results for autocrine+ and exocrine GCNG on MERFISH data

We next tested whether GCNG can be used for applications that utilize predicted interactions as features for downstream analysis. Specifically, we tested whether the outcomes of GCNG can be used as features for assigning function to genes. A popular method for such assignment is Guilt By Association (GBA) [28]. In GBA candidate gene association with known genes is calculated, the total value of which is then used as the final score for this candidate. For this as an alternative to GBA, we trained GCNG to distinguish the spatial expression of pairs of genes within the same function (positive set) from pairs where one gene is associated with that function and the other is not (negative). We focused on functions related to cell-cell communication. In this analysis, we focused on both autocrine+ and exocrine GCNG and applied it to both seqFISH+ cortex and MERFISH datasets. For the functional gene sets, we used GSEA sets [29] for “integrin cell surface interactions,” “cell surface interactions at the vascular wall.” and “cell-cell communication,” which consist of 70 (55), 77 (67), and 79 (66) genes in the seqFISH+ (MERFISH) data, respectively. Performance was evaluated using fivefold cross-validation. Since in function assignment tasks, validation experiments are usually limited to the top few genes, we focused the evaluation on the top 20% predictions based on the scores of GBA and GCNG. Results are presented in Fig. 5 and indicate that for communication-related functions, using spatial information can improve functional gene assignment.

Given its performance on accurately identifying known genes from the GSEA functional sets, we next used GCNG to predict novel functional genes for these three GSEA cell interaction sets using the MERFISH dataset. Additional file 1: Fig. S6 presents the Gene Ontology (GO) analysis [30] for the top 100 predicted genes for each of these functions, showing that several of the top categories by GCNG are related to cell communication. Table 1 lists the top 5 genes predicted for each of these functions. As can be seen, the assignment of 13 of the top 15 predicted genes is supported by recent studies, including all the top five predicted for “cell-cell communication” and “cell surface interactions at the vascular wall.” For example, Serpine1, predicted as the 2nd ranked for integrin, was shown to regulate cell migration using receptor-mediated adhesion [33], and Mdm2 (predicted for cell communication) was shown to relocate to the cell membrane during acute kidney injury-chronic kidney disease [36].

**Table 1:** Top predicted genes for cell communication-related GSEA functional sets

**Cell communication**	
scara3	Scara3 can be translocated to cell surface [31].
thbs1	Thbs1 is an extracellular matrix protein involved in cellular interactions [32].
serpine1	Serpine1 regulates cell migration using receptor-mediated adhesion [33].
ccdc144nl	Ccdc144nl is a protein located in the plasma membrane [34]. See https://www.proteinatlas.org/ENSG00000205212-CCDC144NL/cell for location details. And lncRNA CCDC144NL-AS1 can regulate cell migration [35].
mdm2	Mdm2 relocates to cell membrane during acute kidney injury-chronic kidney disease [36].
**Integrin cell surface interactions**	
ctgf	Ctgf can regulate cell-matrix interaction by binding to cell surface proteins [37].
serpinh1	Serpinh1 can promote cancer cell–platelet interaction [38].
lbr	
plod1	
lamc1	LAMC1 encodes extracellular matrix protein, and regulates cell adhesion, invasion and migration [39].
**Cell surface interactions at the vascular wall**	
serpine1	Serpine1 regulates cell migration using receptor-mediated adhesion [33].
ctgf	Ctgf can regulate cell-matrix interaction by binding to cell surface proteins [37].
serpinh1	Serpinh1 can promote cancer cell–platelet interaction [38].
loxl2	LOXL2 can modulate focal adhesion and tight junction in breast cancer cells [40].
pcolce	Pcolce encodes protein as component of extracellular matrix involved in cellular interactions [41].

## Discussion and conclusion

Gene expression data has been extensively and successfully used to infer interaction between genes, gene regulation and temporal and causal effects [5, 42, 43]. With the recent advances in spatial transcriptomics, such data can now be used to infer pairs of genes involved in cell-cell communication. However, directly converting methods used to infer intra-cellular interactions to methods for inferring extra-cellular interactions is not trivial. The spatial data tends to be very sparse, contains several different cell types, and requires specific decisions about the neighborhoods to consider. Other recent approaches attempted to identify downstream targets of activated ligands using bulk and single cell data [44]. However, unlike GCNG, these methods do not attempt to infer novel direct interactions and are only focused on identifying activated pathways using known interactions.

We presented a supervised GCN approach which can be used to identify new interactions from spatial scRNA-Seq data. GCNs have recently been used in computational biology, though prior applications did not focus on cells but rather on intracellular pathways and utilizing known gene-gene and gene-drug interactions to define the graph structure [45, 46]. For example, Zitnik et al. used GCNs to predict polypharmacy side effects by encoding protein and drug interaction knowledge [45]. In contrast, GCNG is focusing on inferring extra-cellular interactions and can work with a general spatial image for which the specific interactions between cells are not known. It generates a neighborhood graph based on distances between cells and uses it, together with the pairwise expression values for genes to predict interactions between and across cells.

Application of our GCNG method to datasets that provide the highest coverage of genes shows that it can successfully identify known ligand-receptor pairs and that it is much more accurate when compared to prior methods proposed for this or to methods that do not utilize the spatial information. Visualization of some resulting predictions highlights the ability of GCNG to focus on a relevant subset of locations rather than on global correlation. The output of GCNG can also be used as features for downstream analysis including methods for gene function assignment and methods for learning interaction networks.

There are several ways in which GCNG can be improved. First, the choice of the number of convolutional layers to use (which relates to the assumption about the propagation distance of secreted proteins) needs to be better handled to fit the needs of individual datasets. Second, GCNG can focus more on specific cell types rather than on the overall interactions. We expect to address these and other issues in future work. Another important direction is that compared to the intra-cellular case, the extra-cellular interactions between genes might be more complex, including different kinds of functional mechanisms, so we hope future GCNG can model them with more details rather than treating them as an identical case. Furthermore, it is noticed that the mean and median performance for the same method are some different and sometimes the compared prior method is even worse than random guess, which is due to the small sample size of train and test dataset. Finally, the results reported are likely an under-estimate of the performance of the method. Note that we use as the test data the *entire* set of known ligand-receptor pairs. While some are likely active in the tissues we analyzed, many pairs that are listed as “positives” in the test data are not, and so labeling them as “negative” is the actually the correct answer (but is still panelized in our evaluations). More generally, if we had a better ground truth data, we would expect that the results would be much better.

GCNG is implemented in Python and both data and an open source version of the software are available from the supporting website, https://github.com/xiaoyeye/GCNG, and Zenodo [47].

## Methods

### Data used

The two seqFISH+ datasets were downloaded from [16]. These datasets included information from two tissues profiling the expression of 10,000 genes in 913 cells in the mouse cortex and in 2050 cells in mouse olfactory bulb (OB). We normalized the expression data such that expression levels for all genes in each cell sum to the same value, as was previously done [16]. The third is a recent dataset from MERFISH. That data consisted of 10,050 genes in 1368 cells [17]. The counts in seqFISH+ and MERFISH data were processed with the following rule:

$$ {\mathrm{expresion}}_{ij}=\frac{{\mathrm{count}}_{ij}}{\sum \limits_j{\mathrm{count}}_{ij}}\times \mathrm{10,000} $$

where *i* represents cell *i* and *j* represents gene *j*. We also downloaded the cell location files for all the three datasets to generate graph matrices.

### Graph representation for spatial transcriptomics data

To determine the neighbors of each cell, we calculated the Euclidean distance in the image coordinates for all cells and used a distance threshold to select neighbors. The threshold value was selected using 10-fold cross-validation (see “Train and test strategy” section below for details), which for the 2D images, we used seemed to represent the number of neighbors that were in physical contact with the cell (Additional file 1: Tabs. S1&S2). Consider the seqFISH+ cortex data as an example. Given the set of neighbors, we constructed an adjacency matrix size of 913 × 913 (where 913 is the number of cells in the seqFISH+ dataset), which we term *A*. In other words, for the symmetric network, *A*, *A*_*ij*_ = *A*_*ji*_ = 1 if *i* and *j* are neighbors and 0 otherwise. Using the adjacency matrix *A*, the normalized (symmetric) Laplacian matrix *L*_*N*_ is defined as [48]:

$$ {L}_N=I-{D}^{-1/2}A{D}^{-1/2}, $$

where ${D}_{ii}=\sum \limits_j{A}_{ij}$, and *I* is the identity matrix. *L*_*N*_ can be used as a graph matrix. We also tried other graph representation matrices in GCNG. One such method which we adopted first normalizes the adjacency matrix to get *A*_*N*_,

$$ {A}_N={D}^{-1/2}A{D}^{-1/2}, $$

where $$ {D}_{ii}=\sum \limits_j{A}_{ij} $$,

and then computes the normalized Laplacian using the normalized adjacency matrix, *L*_*NN*_,

$$ {L}_{NN}=I-{D}_N^{-\frac{1}{2}}{A}_N{D}_N^{-\frac{1}{2}}, $$

where $$ {D}_{N_{ii}}=\sum \limits_j{A}_{N_{ij}} $$.

Finally, we also tested the following formulation of a graph matrix:

$$ {L}^{\prime }={D}^{\prime -1/2}{A}^{\prime }{D}^{\prime -1/2}, $$

where $$ {D}_{ii}^{\prime }=\sum \limits_j{A}_{ij}^{\prime } $$, and *A*^′^ = *A* + *I*.

In this paper, we use the graph matrix *L*_*NN*_ in the exocrine GCNG, and *L*^′^ in the autocrine+ GCNG model, and diagonal matrix for diagonal GCNG. See Additional file 1 for a detailed discussion about graph representations.

### Labeled data

GCN requires labeled data for supervised training. While the exact set of signaling interactions between cells in the cortex data we studied is unknown, we used as true interactions a curated list of interacting ligands and receptors [20], consisting of 708 ligands, and 691 receptors with 2557 known interactions. Of these, 309 ligands and 481 (involved in 1056 known interactions) are profiled by the seqFISH+ datasets we studied, and 270 ligands, 376 (841 known interactions) are included in the MERFISH dataset. These were used for training and testing. See “Train and test strategy” section below for details on how we define and generate positive and negative examples during training.

### GCNG network architecture

To construct GCNG, we used the python packages of “spektral,” “Keras,” and “Tensorflow.” See Fig. 1a for the architecture of GCNG. GCNG uses two types of input: one encodes the graph structure, *L*_*NN*_ of size of 913× 913 as discussed above, while the second encodes node-specific values, and is generated as the normalized expression of a pair of candidate genes using a matrix of dimension of 913 × 2. GCNG consists of two 32-channel graph convolutional layers, one flatten layer, one 512-dimension dense layer, and one sigmoid function output layer for classification. The graph convolutional layer is defined as:

$$ Z= elu\left( LXW+b\right) $$

where *X* is the expression matrix with dimension of 913 × 2 and *L* is the 913 × 913 graph matrix. *W* is a weight matrix of filters (also termed the convolution kernel) with a size of 2 × 32, where 2 corresponds to the two-dimension gene expression of each node, and 32 represents 32 filters or feature maps. *b* is the bias vector term with a size of 1 × 32. The “*elu*” (exponential linear unit) function is defined as:

$$ elu(x)=\left\{\begin{array}{c}x,x>0\\ {}\alpha \left(\exp (x)-1\right),x\le 0\end{array}\right., $$

where *α* = 1 by default. Here, *Z* represents the embedding vectors of all cell nodes with a size of 913 × 32. Note that we are using two convolutional layers here allowing the method to learn indirect graph relationships as well. Since the impact of secreted proteins can be larger than just direct neighbors, such an approach allows the method to infer interactions that may be missed by only considering direct neighbors.

The first graph convolutional layer combines the two inputs and converts them to embedding vectors for cell nodes of dimension of 913 × 32. The second graph convolutional layer combines the embedding vector of each cell with the one learned for its direct neighbors. The flatten layer then converts the matrices generated by the second layer to a vector using *ReLU* activation function. Finally, a dense layer with one-dimensional output is used to predict the interaction probability based on the *sigmoid* activation function. The activation functions used by the different layers are defined below.

$$ \mathrm{ReLU}\ (x)=\left\{\begin{array}{c}x\  if\ x\ge 0\\ {}0\  if\ x<0\end{array}\right., $$

$$ \mathrm{Sigmoi}{\mathrm{d}}_{\theta }(x)=1/\left(1+{e}^{\theta x}\right). $$

And the objective function for the entire GCNG model is as follows:

$$ F=-\sum \limits_{i=1}^N{y}_i\log \left({GCNG}_{\Theta}(x)\right)+\left(1-{y}_i\right)\log \left(1-{GCNG}_{\Theta}(x)\right), $$

where *i* represents the *i*th sample, *y*_*i*_ represents the label for the *i*th sample, and Θ represents all parameters that need to be optimized in GCNG.

We tested one, two, and three graph convolutional layer networks and determined that two layers network led to the best performance. In addition, we also tried GCNG with cell type information as node attribute using one-hot encoding, distance value as edge attributes, and other GNN architectures including “EdgeconditionConv” model [23] and graph attention model [24], see detailed results for these in Additional file 1: Fig. S1.

### Train and test strategy

We evaluated GCNG’s performance using tenfold cross-validation. Train and test sets were completely separated to avoid information leakage: for each fold, 90% of ligands and 90% of receptors were selected at random for training. Known interactions between the 90% training (10% test) proteins were used as the positive train (test) set and the negative train (test) set was composed of randomly selected ligand-receptor pairs that are not known to interact among the training (test) proteins (on average each ligand only interacts with very few receptors so ∼ 99% of random pairs are expected to be negative). Note that pairs for which one of the proteins was part of the training set while another was in the test set were removed and so all proteins in the test set are never seen in training (Fig. 1b). Early stopping through monitoring validation accuracy was used to avoid overfitting. In addition, 20% of the training set pairs were used as validation set to select the distance threshold used for graph matrix generation (see Additional file 1: Fig. S7 and Additional file 1: Tabs. S1&S2 for details), and the patience epoch number was set as half of the total training epoch number. To evaluate models’ performance, we first calculated the individual area under the receiver operating characteristic curve and the area under the precision recall curve (AUROC/AUPRC) for each ligand and then combined them for the figures presented. In addition, the 40% and 60% quantiles of true positive rate (precision) are calculated along with false positive rate (recall) for AUROC (AUPRC).

## Supplementary Information

**Additional file 1: Supplemental data.** Supplementary data contains supplementary Methods description, a list of supplementary Figures and Tables mentioned in the paper.

**Additional file 2.** Review history.

## Data Availability

*Software availability* The Python software in this paper has been deposited in GitHub and is freely available at https://github.com/xiaoyeye/GCNG and Zenodo [47]. *Data availability* All data, scripts, and instructions required to run GCNG in Python can be found in our support website. All other public data can be found following the pipelines in “Data used” and “Labeled data” in the “Methods” section. Specifically, the two seqFISH+ datasets [16] were downloaded from (https://github.com/CaiGroup/seqFISH-PLUS). The third dataset is a MERFISH dataset [17] downloaded from https://www.pnas.org/content/116/39/19490/tab-figures-data. We used as true interactions a curated list of interacting ligands and receptors [20], which was downloaded from (https://static-content.springer.com/esm/art%3A10.1038%2Fncomms8866/MediaObjects/41467_2015_BFncomms8866_MOESM611_ESM.xlsx). For the functional gene assignment, we downloaded from GSEA [29] the “integrin cell surface interactions” (https://www.gsea-msigdb.org/gsea/msigdb/cards/REACTOME_INTEGRIN_CELL_SURFACE_INTERACTIONS.html), “cell surface interactions at the vascular wall” (https://www.gsea-msigdb.org/gsea/msigdb/cards/REACTOME_CELL_SURFACE_INTERACTIONS_AT_THE_VASCULAR_WALL.html), and “cell-cell communication” (https://www.gsea-msigdb.org/gsea/msigdb/cards/REACTOME_CELL_CELL_COMMUNICATION.html) gene sets, respectively.
