# Supplementary material for: GCNG: graph convolutional networks for inferring gene interaction from spatial transcriptomics data
Source: Genome Biol. 2020 Dec 10;21:300. doi: 10.1186/s13059-020-02214-w (PMC7726911; doi:10.1186/s13059-020-02214-w)
Supplement: Supplementary file 1 — Additional file 1: Supplemental data. Supplementary data contains supplementary Methods description, a list of supplementary Figures and Tables mentioned in the paper. [file 13059_2020_2214_MOESM1_ESM.docx]

**Supplement information of GCNG: Graph convolutional networks for inferring gene interaction from spatial transcriptomics data**

Ye Yuan^1^, Ziv Bar-Joseph^1,2*^

^1^Machine Learning Department, School of Computer Science, Carnegie Mellon University, Pittsburgh, PA 15213, USA. ^2^Computational Biology Department, School of Computer Science, Carnegie Mellon University, Pittsburgh, PA 15213, USA. *e-mail: zivbj@cs.cmu.edu

Contents

[Supplementary Methods: 3](#_Toc54867902)

[Graph matrices calculation 3](#_Toc54867903)

[Supplementary Figures: 5](#_Toc54867904)

[Fig. S1 Comparisons to GCNG models with permutation, with cell type information as node attribute, distance value as edge attributes and other GNN architectures including EdgeconditionConv model and graph attention model 5](#_Toc54867905)

[Fig. S2 Detailed AUROC and AUPRC for Fig. 2A&B 6](#_Toc54867906)

[Fig. S3 Detailed AUROC and AUPRC for Fig. 2C&D 7](#_Toc54867907)

[Fig. S4 Whole plots of typical gene pair’s spatial expression pattern for Fig. 3 8](#_Toc54867908)

[Fig. S5 Cell type spatial distribution 9](#_Toc54867909)

[Fig. S6 GO terms based on top 100 predicted unknown genes for cell communication related GSEA functional sets 10](#_Toc54867910)

[Fig. S7 Distance threshold selection based on the validation set in 10-fold cross validation 11](#_Toc54867911)

[Fig. S8 Results for seqFISH+ OB data 12](#_Toc54867912)

[Supplementary Tables: 13](#_Toc54867913)

[Tab. S1 13](#_Toc54867914)

[Tab. S2 13](#_Toc54867915)

[Reference: 13](#_Toc54867916)

# Supplementary Methods:

## Graph matrices calculation

The normalized (symmetric) Laplacian matrix $\boldsymbol{L}_{\boldsymbol{N}}$was generated by setting:

$L_{N}=I-D^{-1/2}AD^{-1/2}$, Where $D_{ii}=\sum_{j} A_{ij},$ *I* is the identity matrix. (1)

$\boldsymbol{L}_{\boldsymbol{N}}$ can be the operator approximation of 1^st^ order Chebyshev polynomials approximation (CPA) for original spectral convolutions on graphs [1], with the assumption that its zero order is the opposite number of its 1^st^ order coefficient [2]. Specifically, the spectral convolutions on graphs is defined as $g_{\theta}*x=Ug_{\theta}(\Lambda)U^{T}x$, where *U* is matrix of eigenvectors of normalized Laplacian matrix $\boldsymbol{L}_{\boldsymbol{N}}$ from *A* (Eq. 1), *Λ* is diagonal matrix of eigenvalues of $\boldsymbol{L}_{\boldsymbol{N}}$, $U^{T}x$ is the graph Fourier transform of $x$, $g_{\theta}$ is a function of *Λ*. The K^th^ order CPA $g_{\theta^{'}}\left( \Lambda\right)\approx\sum_{k=0}^{K} \theta_{k}^{'}T_{k}\left( \tilde{\Lambda} \right)$ was then used to approximate $g_{\theta}(\Lambda)$, where $\tilde{\Lambda}=2\Lambda/ \lambda_{max}-I_{N}$, $\lambda_{max}$ is the largest entry of $\Lambda$, $T_{k}\left( x \right)=2xT_{k-1}\left( x \right)-T_{k-2}(x)$, $T_{0}\left( x \right)=1 \mathrm{and} T_{1}\left( x \right)=x$. The 1^st^ order CPA can be approximated as $\theta_{0}x-\theta_{1}D^{-1/2}AD^{-1/2}x$ with $\lambda_{max}\approx2$. By further assumption of ${\theta= \theta}_{0}=\theta_{1}$, the final approximation becomes:

$g_{\theta}*x\approx\theta(I-D^{-1/2}AD^{-1/2})x$ (2)

We also tried two alternative graph matrix approximation. a) The normalized Laplacian matrix can be generated from a normalized adjacent matrix, ***A_N_*** instead of ***A****:*

$A_{N}=D^{-1/2}AD^{-1/2}$, Where $D_{ii}=\sum_{j} A_{ij}$. (3)

Then the normalized (symmetric) Laplacian matrix $\boldsymbol{L}_{\boldsymbol{NN}}$from ***A_N_*** was generated as the graph matrix:

$L_{NN}=I-D_{N}^{-1/2}A_{N}D_{N}^{-1/2}$, Where $D_{N_{ii}}=\sum_{j} A_{N_{ij}}$. (4)

b) Following the same assumption of $\theta= \theta_{0}={-\theta}_{1}$ in ref [2], and renormalized trick of $I+ D^{-1/2}AD^{-1/2}\to D^{'-1/2}A^{'}D^{'-1/2}$, the convolutional approximation becomes $g_{\theta}^{'}*x\approx\theta D^{'-1/2}A^{'}D^{'-1/2}x$, Where $D_{ii}^{'}=\sum_{j} A_{ij}^{'}$, and $A^{'}=A+I$. Then, graph matrix approximation can be generated by setting, $L^{'}=D^{'-1/2}A^{'}D^{'-1/2}$, Where $D_{ii}^{'}=\sum_{j} A_{ij}^{'}$, and $A^{'}=A+I$. Here we use the graph matrix $L_{NN}$ in the exocrine GCNG and $L^{'}$ in the autocrine+ GCNG model, and diagonal matrix for diagonal GCNG.

# Supplementary Figures:

## Fig. S1 Comparisons to GCNG models with permutation, with cell type information as node attribute, distance value as edge attributes and other GNN architectures including EdgeconditionConv model and graph attention model


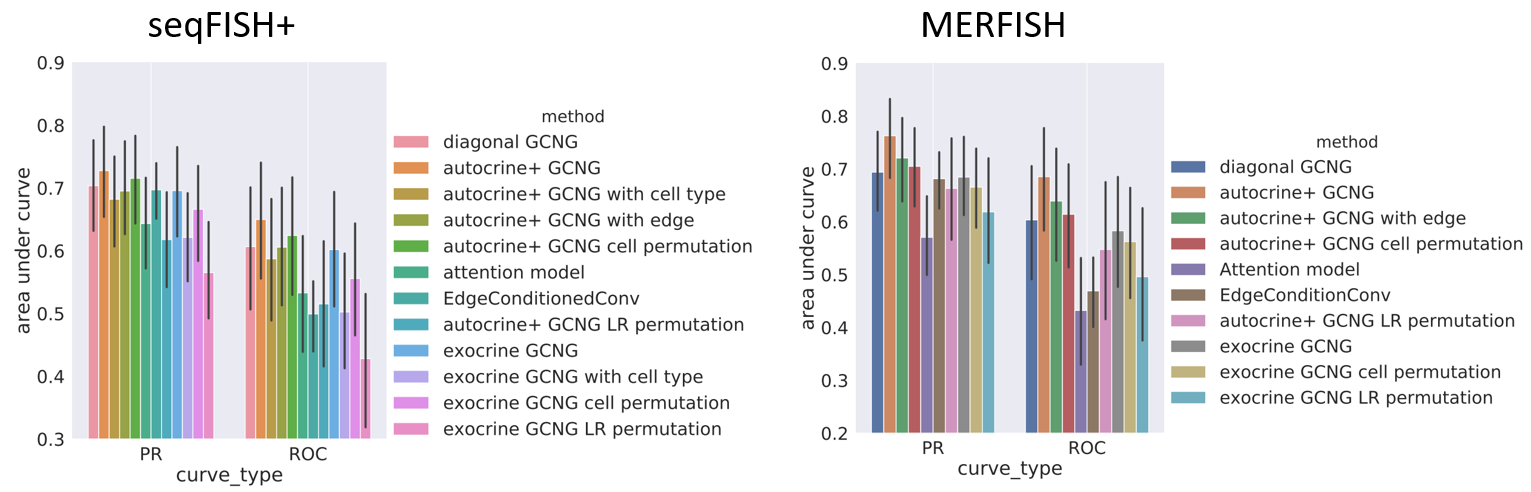


Here exocrine GCNG model is GCNG that only has exocrine gene interactions; diagonal GCNG represents model with only diagonal graph matrix; autocrine+ GCNG represents GCNG with both autocrine and exocrine interactions. For GCNG with cell type, we added the one-hot encoding cell type information for each cell node in the graph. For GCNG with edge, we substituted the distance matrix directly into the equations of autocrine+ GCNG model. For GCNG with cell permutation, we permuted all cells position index while kept the original graph matrix to break the neighborhood relationship among cells. For GCNG with LR permutation, we permuted the set of interacting ligand-receptor pairs while kept the degree and ligands and receptors fixed. For EdgeConditionedConv model, we called the “EdgeConditionedConv” layer function in Python “spektral” package. For Attention model, we called “GraphAttention” layer function in Python “spektral” package.

## Fig. S2 Detailed AUROC and AUPRC for Fig. 2A&B


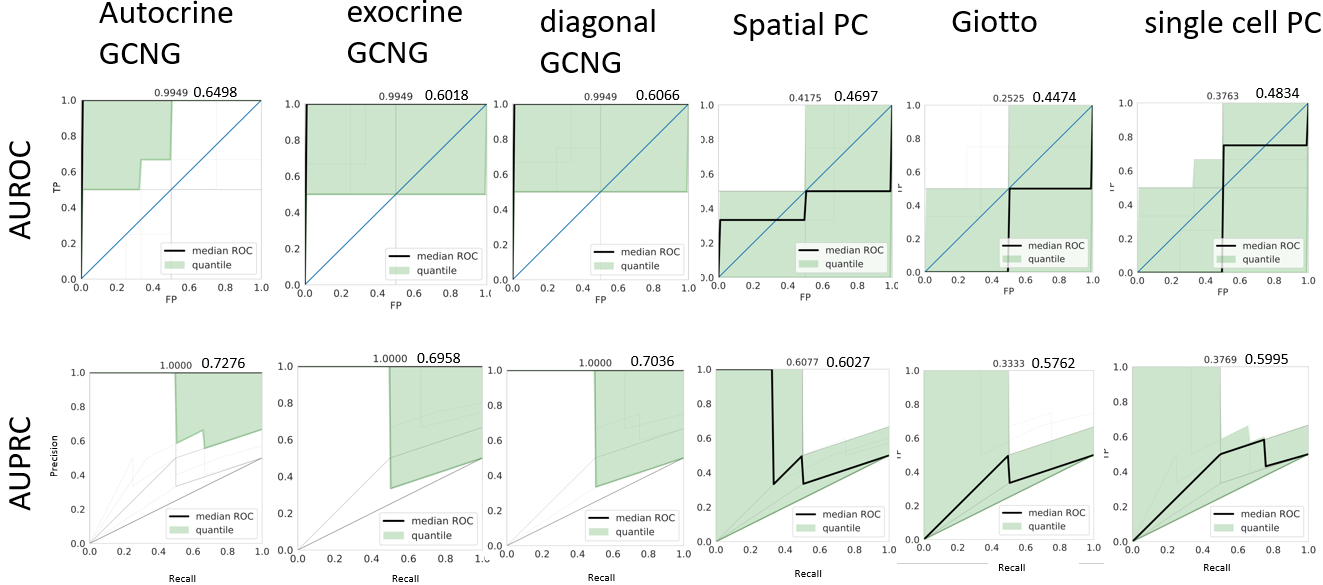


Here each gray line represents one ligand (in total 91 ligands), black line represents the median curve, and the light green part represents the region between 40-60 quantile. Mean and median of area under the curves are shown in top of each panel.

## Fig. S3 Detailed AUROC and AUPRC for Fig. 2C&D


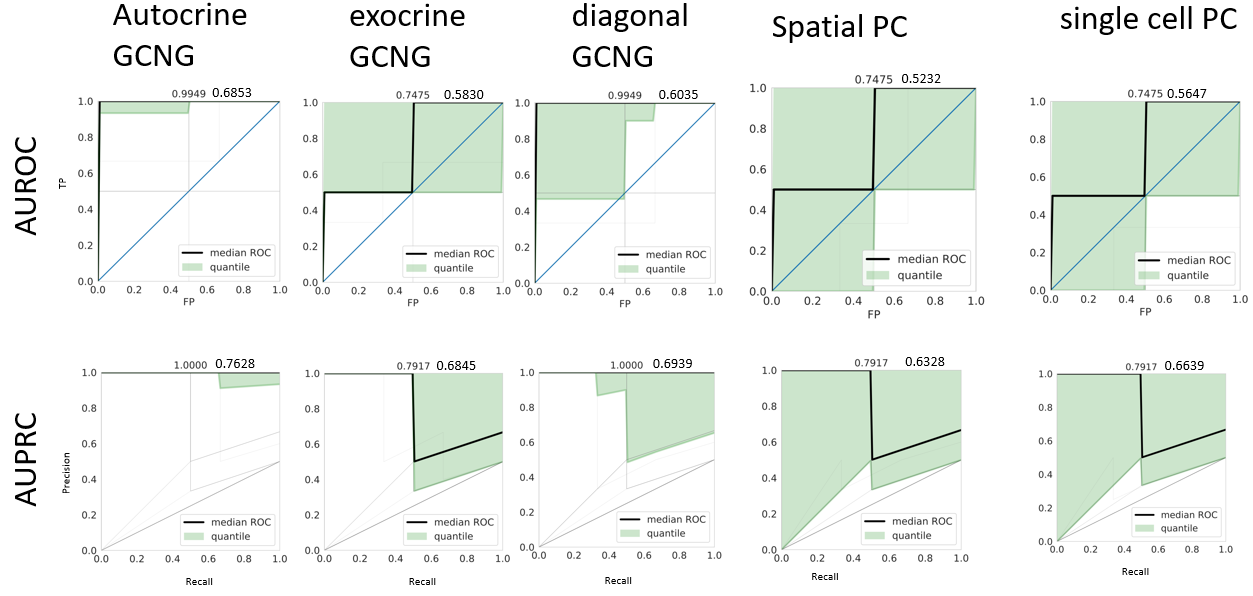


Here each gray line represents one ligand (in total 73 ligands), black line represents the median curve, and the light green part represents the region between 40-60 quantile. Mean and median of area under the curves are shown in top of each panel.

## Fig. S4 Whole plots of typical gene pair’s spatial expression pattern for Fig. 3


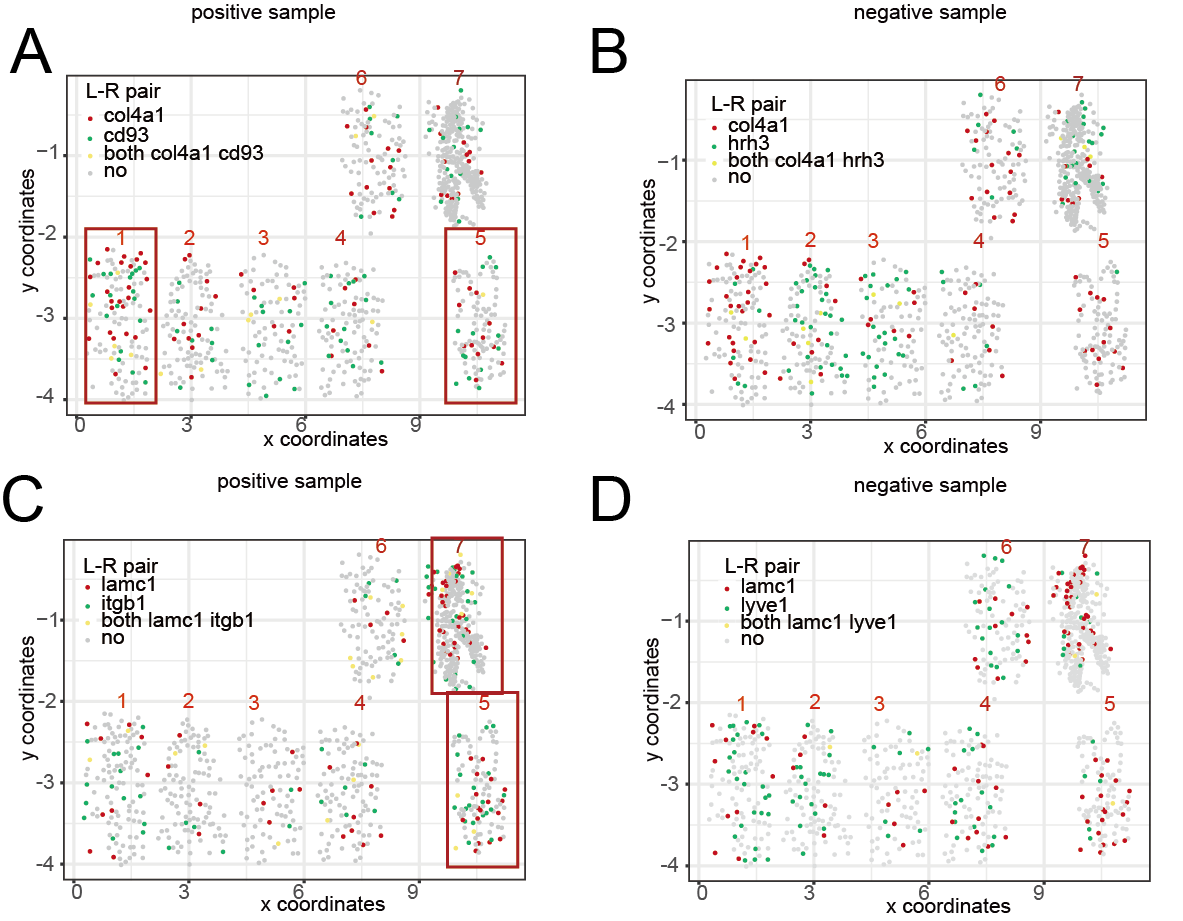


**(A, B)** The spatial expression distribution of correctly predicted positive (cd93) and negative (hrh3) samples for ligand col4a1. Cells highly expressing col4a1 (red) and cd93 (green) are both concentrated in the 1^st^ and 5^th^ fields as shown in the red boxes (Fig. 3). **(C, D)** The spatial expression distribution of correctly predicted positive (itgb1) and negative (lyve1) samples for ligand lamc1. Cells highly expressing lamc1 and itgb1 are both concentrated in the 5^th^ and 7^th^ fields as shown in the red boxes.

## Fig. S5 Cell type spatial distribution

**
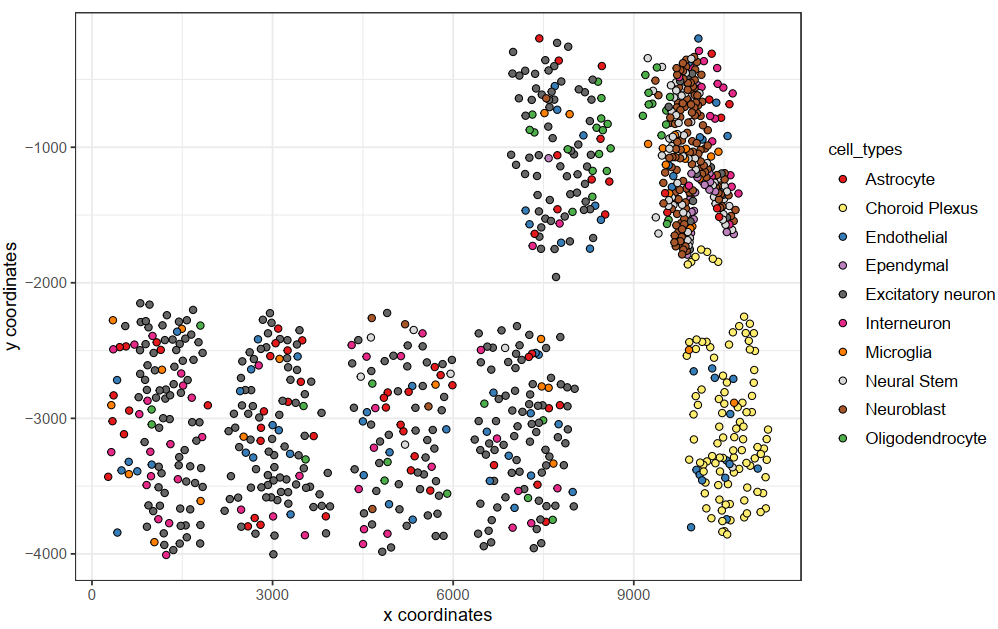
**

This Figure is generated directly by Giotto using the data it provided [3].

## Fig. S6 GO terms based on top 100 predicted unknown genes for cell communication related GSEA functional gene sets


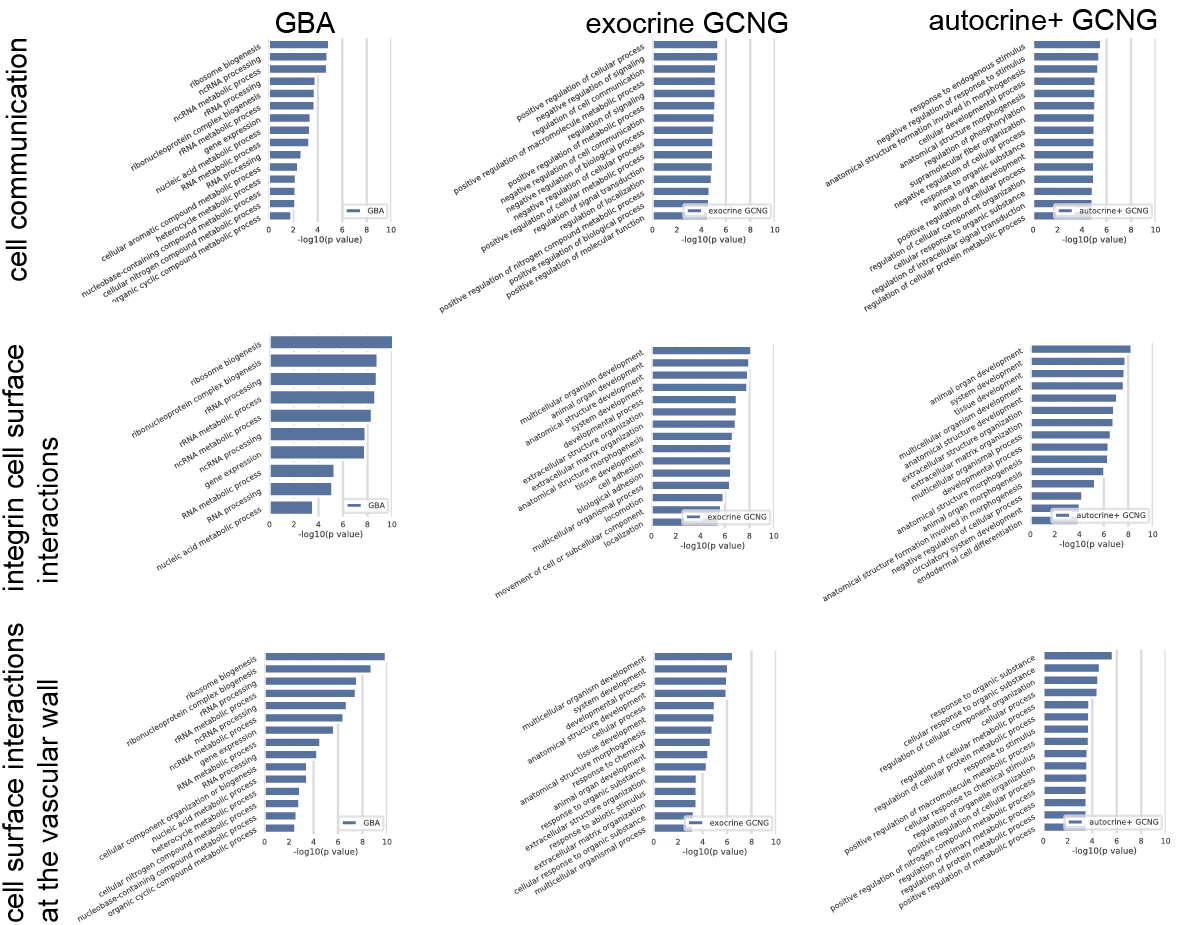


Here we used GBA and GCNG methods to predict possible functional genes among all unknown genes excluding the known GSEA functional genes based on MERFISH data, and selected the top 100 predicted genes for GO term analysis. As can be seen, GCNG models give several top GO terms related to cell communication for all the three GSEA functions, such as ’regulation of cell communication’, ‘multicellular organism development’, and ‘extracellular structure organization’, while GBA method does not. In Tab. 1, the top genes are predicted by exocrine GCNG.

## Fig. S7 Distance threshold selection based on the validation set in 10-fold cross validation


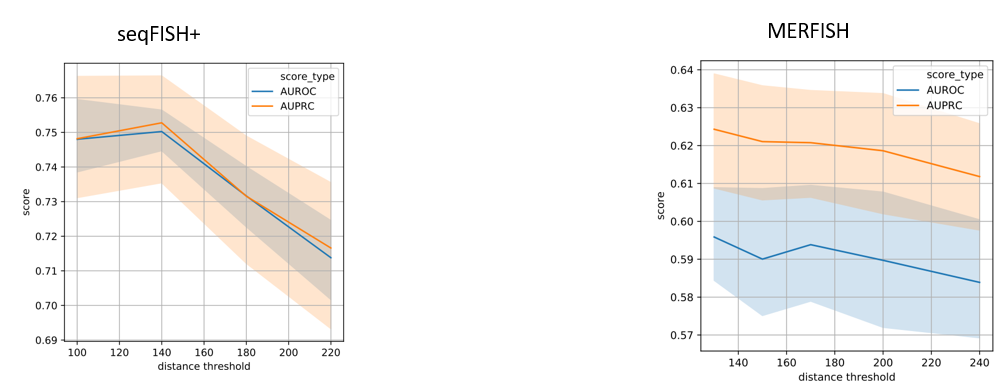


Here we used 10-fold cross validation to select the best distance threshold. We regarded the distance threshold as a hyperparameter and learn it as part of the training process. This is done by dividing the training data intro a training set (used to learn parameters) and a validation set (used to set hyper-parameters). Thus, each threshold is learned independently for each cross validation run and is not based on the entire dataset. And the threshold was selected as 140, and 130 for seqFISH+ and MERFISH data respectively.

## Fig. S8 Results for seqFISH+ OB data


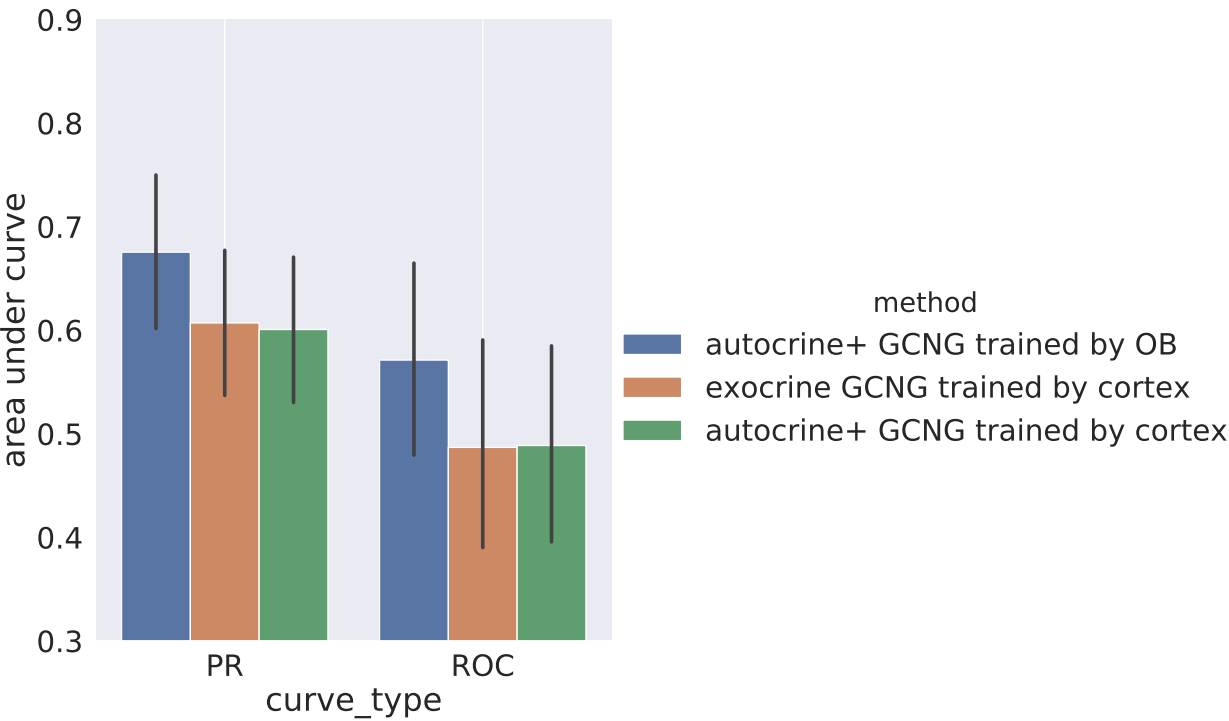


Here we used GCNG models trained by seqFISH+ cortex dataset with 913 cells to predict the test gene pairs on seqFISH+ OB data. OB data has 2,050 cells, so we firstly cut the data into 913 cells and then fed it into the cortex trained GCNG models. As can be seen, the model trained by OB training data is better than those trained by cortex data.

# Supplementary Tables:

## Tab. S1

**Average cell neighborhood for different distance threshold for seqFISH+ cortex data**


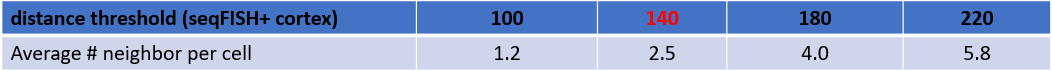


The red number represent the threshold with the best validation performance.

## Tab. S2

**Average cell neighborhood for different distance threshold for MERFISH data**


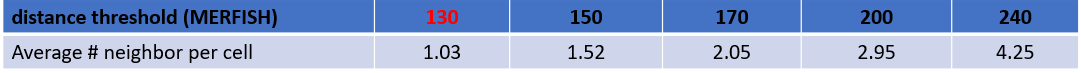


The red number represent the threshold with the best validation performance.

# Reference:

1. Defferrard M, Bresson X, Vandergheynst P, editors. Convolutional neural networks on graphs with fast localized spectral filtering. Advances in neural information processing systems; 2016.

2. Kipf TN, Welling M. Semi-supervised classification with graph convolutional networks. arXiv preprint arXiv:02907. 2016.

3. Dries R, Zhu Q, Eng C, Sarkar A, Bao F, George R, et al. Giotto, a pipeline for integrative analysis and visualization of single-cell spatial transcriptomic data. bioRxiv. 2019.
