# Supplementary material for: GCNG: graph convolutional networks for inferring gene interaction from spatial transcriptomics data
Source: Genome Biol. 2020 Dec 10;21:300. doi: 10.1186/s13059-020-02214-w (PMC7726911; doi:10.1186/s13059-020-02214-w)
Supplement: Supplementary file 2 — Additional file 2. Review history. [file 13059_2020_2214_MOESM2_ESM.docx]

**1^st^ round**

**Reviewer 1**

The authors proposed a supervised method for identifying cell-cell interactions using the Graph Convolution Neural network approach, and performed cross validations mainly on one dataset. The authors showed that their approach performed better than other existing methods. The method is sound. However, there is no new biology learned from the approach. Thus, the manuscript is better suited in a more Bioinformatics focused journal.

Here are some concerns:

1) There is no gold standard for cell-cell interactions. Interactions identified could be random noise if there is no biological follow-up to validate them.

2) There are many parameters to choose/optimize so that an independent dataset is required to access its performance.

3) some underlying assumptions are not correct. For example, autocrine is a significant part of cell regulation. In the GCN model, the diagonal cells in the adjacency matrix are required to be zero, which exclude all autocrine regulations.

4) One of important biological questions is which type of cells interact with which type of cells under which condition. The model treats all cells equally, which is far from true biology.

**Reviewer 2**

The authors may want to consider the following suggestions for a revision:

- Detailed description of the dataset should be provided

- The proposed model is too simple. There are a lot of improvement can be made. For example:

+ Adding node description: since the distance between node is different, adding this information as edges information can help increase accuracy.

+ Replace graph convolution with attention graph convolution. This can help to determine the effect of certain cell (ligand-receptor pair) to other ligand-receptor interactions.

- The performance of the model is not very good. Using the output of the model for downstream tasks is not a good idea due to error propagation.

- Other questions:

+ The model is based on a rigid map of cells, and it can predict new ligand-receptor interactions on a cell list of 913 cells. How will we use it when we want to check a novel ligand-receptor interaction in a new set of cells?

+ How did you normalize your data, especially for your test dataset?

+ The threshold was determined solely by using the training set or by using all the data? Did you use the same threshold for training and testing?

+ Please describe clearly the primary evaluation metric which was used to optimize your model. Why did you choose that metric?

+ Did you apply any technique to handle imbalanced data?

+ When testing, is there any link between a sample in the training set and another sample in the test set?

These questions need to be clarified and a major revision is needed for this manuscript.

**Author response**

Reviewer 1:

The authors proposed a supervised method for identifying cell-cell interactions using the Graph Convolution Neural network approach, and performed cross validations mainly on one dataset. The authors showed that their approach performed better than other existing methods. The method is sound. However, there is no new biology learned from the approach. Thus, the manuscript is better suited in a more Bioinformatics focused journal.

We note that this is submitted as a ‘Methods’ paper. Genome Biology’s guidelines for such papers state the following ‘Method articles should describe novel methods that are shown to be a clear advance over existing state-of-the-art methods. … A Method article need not necessarily provide novel biological insights, but these can help to demonstrate the method’s utility.’

As the reviewer notes, we developed a novel method for a new data type and analyzed real data which demonstrated the usefulness of GCNG when compared to current state of the art approaches, which is the main requirement for a Method paper. Still, based on this comment we extended the biological analysis in a number of ways in the revised version. We added an analysis for the MERFISH dataset with 1,368 cultured U- 2 OS cells, discussing both ligand-receptor interactions, causality inference and functional gene assignment (revised Figs. 2, 4 and 5). In addition, we now analyze another seqFISH+ dataset profiled in mouse olfactory bulb. Furthermore, we expanded the method by including the use of an autocrine plus model which enables the identification of self regulation interactions and have updated the novel predicted genegene interactions using results from this model.

Here are some concerns:

1) There is no gold standard for cell-cell interactions. Interactions identified could be random noise if there is no biological follow-up to validate them.

We agree that for the specific tissues and cells we looked at there is little ground truth that can be used to statistically determine the accuracy of the results. In the original submission we attempted to address this by selecting a subset of the predictions and describing their likely relevance to the tissues being studied. To perform a larger scale statistical tests, as the reviewer requests, we further extended our analysis to determine whether the interactions identified are indeed the result of noise or represent real ligand-receptor interactions for these cells. For this, we randomized several aspects of the data and compared the results obtained from the random dataset to the results of the real data. If our pairs are really only noise we would expect to find a similar number of pairs when using the random data. As we show, this is not the case. Specifically, when we use GCNG on a permuted dataset (in which we permute the neighbors of each cell) we see a large drop in performance (for example mean AUROC for MERFISH, in real VS random drops from 0.69 to 0.61, see Fig. S3 for more comparisons). Note that in these permutations we only change the neighborhood assignment and so autocrine interactions are still used in the random as well. We also tested another randomization strategy in which we permute the set of interacting ligand-receptor pairs used for training and testing. Results for this analysis again support the notion that GCNG identifies real interactions (for example mean AUROC for seqFISH+ data, in real VS random drops from 0.65 to 0.52 (Fig. S3)). We have added these results to the Supplement (Fig. S3).

2) There are many parameters to choose/optimize so that an independent dataset is required to access its performance.

We agree that parameter setting is a major issue for any deep learning method. To select such parameters we relied on the long established method, cross validation in which a training set is used to learn parameters for the network, a validation set is used to select hyper-parameters (size and distance used for constructing neighborhood graph) and a test set is used for evaluations (all in a cross validation setup).

Specifically, we divided all ligand-receptor pairs into train, validation and test sets. These were kept strictly separate such that a ligand / receptor can only appear in *one of these sets* (so we cannot have a pair A->B in the training set and a pair A->C in the test set for three proteins, A, B and C). Thus, all parameter selections for all datasets are fully learned and do not require any prior knowledge or arbitrary decisions. See Methods and Fig. S5 for complete details on the parameter learning process.

3) some underlying assumptions are not correct. For example, autocrine is a significant part of cell regulation. In the GCN model, the diagonal cells in the adjacency matrix are required to be zero, which exclude all autocrine regulations.

We thank the reviewer for this excellent comment. Based on this we revised the method to allow for endocrine relationships. As the reviewer suspected, such model leads to even better results than the zero diagonal model we used in the original submission. We now present results in the paper for three possible models: diagonal GCNG with only autocrine interactions, exocrine GCNG with only exocrine interactions, and autocrine plus GCNG with both autocrine and exocrine interactions. Our results indicate that the third works best, but users are free to use any model they want (for example, those users that are only interested in cell-cell interactions may want to only use the exocrine option). Full comparisons of all models are presented in Fig. 2 and Fig. S3.

4) One of important biological questions is which type of cells interact with which type of cells under which condition. The model treats all cells equally, which is far from true biology.

We agree that general cell type interactions are of great interest. But these are not the only questions researchers ask. For example, the new MERFISH data we analyze (from U-2 OS cells) only profiles a single cell type and there the focus is on the *proteins* involved in the interactions between cells of the same type rather than interactions between different types of cells. Still, based on this comment we extended GCNG to enable it to use cell type information as well. This is done by expanding the encoding of every node (cell) using one-hot encoded cell type information. We used the expanded graph representation to analyze the seqFISH+ cortex data. However, at least for this data cell type information did not help improve the performance. We further investigated this and as the spatial pattern in Fig.3 indicates we sometimes see similar interactions between cells of different types (same pair of proteins but different cell types). Still, even though we were not successful in using cell type information for the datasets we analyzed this option is now available to users of GCNG and they can decide whether they would like to use an encoding that utilizes this information or not.

Reviewer #2

The authors may want to consider the following suggestions for a revision:

- Detailed description of the dataset should be provided

As suggested we have added detailed descriptions of the datasets in Methods.

- The proposed model is too simple. There are a lot of improvement can be made. For example:

+ Adding node description: since the distance between node is different, adding this information as edges information can help increase accuracy.

+ Replace graph convolution with attention graph convolution. This can help to determine the effect of certain cell (ligand-receptor pair) to other ligand-receptor interactions.

As suggested we have implemented several extensions to the proposed model including the ones suggested by the reviewer. These include:

1) A new autocrine plus model where we allow both self and external cell-cell interactions. As we show, this model actually improves upon the original models we used in the initial submission that did not allow for self interactions.

2) A revised model in which we expand the node information to encode the assigned cell type for each cell. This is done using one-hot encoding to add the cell type information. As Fig. S3 shows, such extension did not improve results for the data we used, though could be useful for other datasets.

3) A revised model where we include a continuous value to represent the actual distance between cells (edge information). We have tested two variants for adding such information. The first encoded the distance matrix as a direct input to the GCN layer function and the second used the EdgeConditionedConv method (Simonovsky & Komodakis, 2017, CVPR). Results are presented in Fig. S3. As the figures show, for the data we analyzed such encodings did not improve results though this may be attributed to the size of the data. The continuous encodings of edge distance lead to more complexity for the model which in turn can lead to overfitting for smaller datasets. Still, in the revised version we used a new strategy to select distances based on an additional validation set. While the final graph we use is still binary (i.e. an edge either exists or does not) by using the validation strategy we allow the method to determine the best distance to focus on which indeed improves on the original results. See Fig. S5 for details.

4) We also tested the graph attention model the reviewer suggested by calling the ‘GraphAttention’ layer function in Python’s ‘spektral’ package’ (Velickovic et al.,2018, ICLR). However, as the results in Fig. S3 show, this method did not improve the result.

- The performance of the model is not very good. Using the output of the model for downstream tasks is not a good idea due to error propagation.

The results reported are likely an under-estimate of the performance of the method. Note that we use as the test data the *entire* set of known ligand-receptor pairs. While some are likely active in the tissues we analyzed, many pairs that are listed as ‘positive’ in the test data are not, and so labeling them as ‘negative’ is the actually the correct answer (but is still panelized in our evaluations). Moe generally, if we had a better ground truth data we would expect that the results would be much better (an anecdotal indication that this is indeed the case is the list of top predicted pairs that we show, several of which are known to interact in the tissues we analyze). Thus, whether down-stream analysis can be performed or not is really up to the user.

We note that the current version further improves on the original results we presented by using the autocrine model so this provides another support to the use of these results in down-stream analysis. We have now revised the Discussion to emphasize this point and to mention possible

issues with using the predictions in down-stream analysis.

- Other questions:

+ The model is based on a rigid map of cells, and it can predict new ligand-receptor interactions on a cell list of 913 cells. How will we use it when we want to check a novel ligand-receptor interaction in a new set of cells?

This depends on the similarity of the experiments. In the current paper we analyze data from three different technologies / tissues and using models learned based on one of them to predict for another does not work very well (see Fig. S4). However, the data we use to train the model is context independent (i.e. general interactions of ligand and receptors) and so in theory it can be used for any set of cells. Once trained the model can be applied to gene pairs not used in the training and would predict new interactions as we showed in Tab. 1.

+ How did you normalize your data, especially for your test dataset?

We followed Eng C-HL et al. (PMID: 30911168). Briefly, we normalized each dataset assuming equal total expression level in each cell (though obviously different values for different genes).

+ The threshold was determined solely by using the training set or by using all the data? Did you use the same threshold for training and testing?

We regard the distance threshold as a hyperparameter and learn it as part of the training process. This is done by dividing the training data into a training set (used to learn parameters) and a validation set (used to set hyperparameters). Thus, each threshold is learned independently for each cross validation run and is not based on the entire dataset (a clear separation between training and test data). Please see Fig. S5 for detailed results.

+ Please describe clearly the primary evaluation metric which was used to optimize your model. Why did you choose that metric?

The primary evaluation metric is cross entropy since the last layer is exactly the logistic regression. We now added the optimization function description in Methods section, which is

F= -Σy_i *log(GCNG_Θ (x))+(1-y_i)*log(1-GCNG_Θ (x)),

Where i represents the ith sample, y_i represents the label for the ith sample and Θ represents all parameters that need to be optimized in GCNG.

+ Did you apply any technique to handle imbalanced data?

Yes, we selected training and test negative pairs such that the ratio is equal for both, this is also reflected in the ROC curves we present. We now explicitly mention this in

Methods.

+ When testing, is there any link between a sample in the training set and another sample in the test set?

This is an important point which we actually took care to avoid. As shown in Fig. 1B, to avoid information leakage, we completely separate the training, validation and test sets for each CV round. Thus, each protein (either a ligand or receptor) can only be present in *one* of these sets (for example, we cannot use A->B as a pair in the training and A- >C as a pair in the test for three proteins, A,B and C).

**2^nd^ round**

**Reviewer 1**

The authors reasonably addressed my concerns, especially about model assumptions about autocrine regulations.

With regard to biological insight, it is still hard to judge any biological insights that are derived from the analysis. The evidences cited in Table 1 including co-clustering in cancers do not seem support any interactions.

**Reviewer 2**

I am generally OK with the response from the authors. However, a minor revision is needed so that the authors can include more biology discussion into the manuscript.

In fact, there is no new technical contribution in the proposed framework. However, application of GCNN in this topic is rather new. So the manuscript may be of interest for readers of this journal.

**Author response**

In this version we addressed the remaining minor comments and made the changes requested to the title and abstract.

Specifically, we added additional biological analysis in which we used GCNG to predict new cell-cell interaction proteins. We also added a new table, Table 1, in which we list some of the novel top predictions for these functions.
